# Correlations between local peripapillary choroidal thickness and axial length, optic disc tilt, and papillo-macular position in young healthy eyes

**DOI:** 10.1371/journal.pone.0186453

**Published:** 2017-10-12

**Authors:** Takehiro Yamashita, Taiji Sakamoto, Naoya Yoshihara, Hiroto Terasaki, Minoru Tanaka, Yuya Kii, Kumiko Nakao

**Affiliations:** Department of Ophthalmology, Kagoshima University Graduate School of Medical and Dental Sciences, Kagoshima, Japan; International University of Health and Welfare, JAPAN

## Abstract

Optical coherence tomography (OCT) has made it possible for clinicians to measure the peripapillary choroidal thickness (ppCT) noninvasively in various ocular diseases. However, the ocular factors associated with the ppCT have not been conclusively determined. The purpose of this study was to determine the relationship between the local ppCT and the axial length, optic disc tilt, and the angle of the papillo-macular position (PMP) in healthy eyes. This was a prospective, observational cross-sectional study of 119 right eyes of 119 healthy Japanese volunteers. The ppCT was manually measured at eight sectors around the optic disc using the B-scan images of the Topcon 3D OCT RNFL 3.4 mm circle scan. The trajectory of the retinal pigment epithelium in the B-scan image was fitted to a sine curve using ImageJ, and the amplitude of the sine curve was used to determine the degree of the optic disc tilt. The PMP angle was determined in the color fundus photographs. The relationships between the ppCT and the axial length, the optic disc tilt, and PMP angle were determined by Spearman and multiple correlation analyses. The mean age was 25.8 ± 3.9 years and the mean axial length was 25.5 ± 1.4 mm. The ppCT was significantly and negatively associated with the axial length (R = -0.43 to -0.24, *P*<0.01) and positively associated with the PMP angle (R = 0.28 to 0.37, *P*<0.01) in all eight circumpapillary sectors. The temporal and infratemporal ppCTs were significantly and negatively associated with the optic disc tilt (R = -0.31, -0.20, *P*<0.05). The results of multiple regression analyses were similar to that of Spearman correlation analysis. In conclusion, the axial length and PMP angle can affect the ppCT in all circumferential sectors, however the tilt of the optic disc is correlated with only some of the sectors. This should be remembered in interpreting the ppCT.

## Introduction

Optical coherence tomography (OCT) is a noninvasive imaging technique that can be used to determine the morphology of the retina, choroid, and optic disc with micrometer resolution [1]. The images obtained by OCT can be used to quantify the thickness of the different retinal and choroidal layers which can be used to assess the pathophysiology of normal and diseased eyes. Importantly, the images can be recorded noninvasively with good repeatability and reproducibility [2–11].

OCT has been used to measure the peripapillary choroidal thickness (ppCT) in various ocular diseases [12–25]. Because the peripapillary area is believed to be the area most affected in glaucoma and myopia, the ppCT has been studied extensively in these ocular conditions. However, there is not yet conclusive agreement in the changes in the ppCT in various diseases. For example, Lin et al [21] reported that the ppCT is decreased in eyes with glaucoma, while Wang et al [13] reported that the ppCT is not altered in eyes with glaucoma.

The earlier studies showed that the average ppCT was significantly thinner in eyes with longer axial lengths, lower intraocular pressure, flatter cornea, larger parapapillary alpha and beta zones, poorer visual acuity, a posterior staphyloma, retinochoroidal atrophy, and tilted optic disc [14, 15, 26]. In these studies, the ppCT was expressed as the average of the ppCT of all 8 sectors of the circumferential peripapillary area. However, using the average of the ppCT alone can miss focal changes even though focal changes might be more meaningful. Logically, the ppCT should be affected not only by systemic factors, but also by local factors such as a tilting of the optic nerve head (ONH) which could deform the peripapillary tissue. This could then lead to impairments of the corresponding area of the visual field.

Thus, the purpose of this study was to determine whether local changes of the ppCT were significantly correlated with the axial length, optic disc tilt, and papillo-macular position (PMP) angle. To accomplish this, we measured the axial length of the eye by ultrasonography, the optic disc tilt by a new method [26–27], and the PMP angle in the fundus photographs. We then determined the association of these factors with the ppCT of each sector of the optic disc.

## Methods

All of the procedures used conformed to the tenets of the Declaration of Helsinki. A written informed consent was obtained from all of the subjects after an explanation of the procedures to be used. The study was approved by the Ethics Committee of Kagoshima University Hospital, and it was registered with the University Hospital Medical Network (UMIN)-clinical trials registry. The registration title was, “Morphological analysis of the optic disc and the retinal nerve fiber in myopic eyes” and the registration number was UMIN000006040. A detailed protocol is available at https://upload.umin.ac.jp/cgi-open-bin/ctr/ctr.cgi?function=brows&action=brows&type=summary&recptno=R000007154&language=J.

## Subjects

### Ethics statement

This was a cross-sectional, observational study. We initially examined 133 eyes of 133 volunteers who were enrolled between November 1, 2010 and February 29, 2012. The volunteers had no known eye diseases as determined by examining their medical charts, and the data from only the right eyes were analyzed. The eligibility criteria were: age ≥20 years but ≤40 years; eyes normal by slit–lamp biomicroscopy, ophthalmoscopy, and OCT; best-corrected visual acuity (BCVA) ≤0.1 logarithm of the minimum angle of resolution (logMAR) units; and intraocular pressure (IOP) ≤21 mmHg. The exclusion criteria were: eyes with known ocular diseases such as glaucoma, presence of a staphyloma, optic disc anomalies; presence of visual field defects; and prior refractive or intraocular surgery. The presence of a staphyloma was determined by B-mode echo ultrasonography. Eyes with poor OCT image quality caused by poor fixation were excluded.

### Measurement of axial length and refractive error

All eyes had a standard ocular examination as reported elsewhere [27, 28] including; slit-lamp biomicroscopy of the anterior segment, ophthalmoscopy of the ocular fundus, pneumo-tonometric (CT-80, Topcon, Tokyo, Japan) measurements of the IOP, and AL-2000 ultrasonographic (TOMEY, Nagoya, Japan) measurements of the axial length. The refractive error (spherical equivalent) was measured with the Topcon KR8800 autorefractometer/keratometer.

### Measurements of peripapillary choroidal thickness (ppCT)

All eyes were examined by a single examiner (TY). The retinal nerve fiber layer thickness (RNFLT) was measured with the TOPCON 3D OCT-1000 MARK II using the RNFL 3.4 mm circle scan. In this protocol, 1024 A-scans/circle, 16 overlapping B-scans/image, and direct B-scan observations were recorded. The OCT images and the color fundus photographs were recorded at the same time. The optical system of the OCT instrument detected the edge of the optic disc in the fundus image, and the scan circle was automatically centered on the optic disc just before the OCT image was recorded. To exclude errors in the scan circle centration, one examiner (YK) checked offline that the center of the scan circle was located at the center of the optic disc.

**Fig 1:**
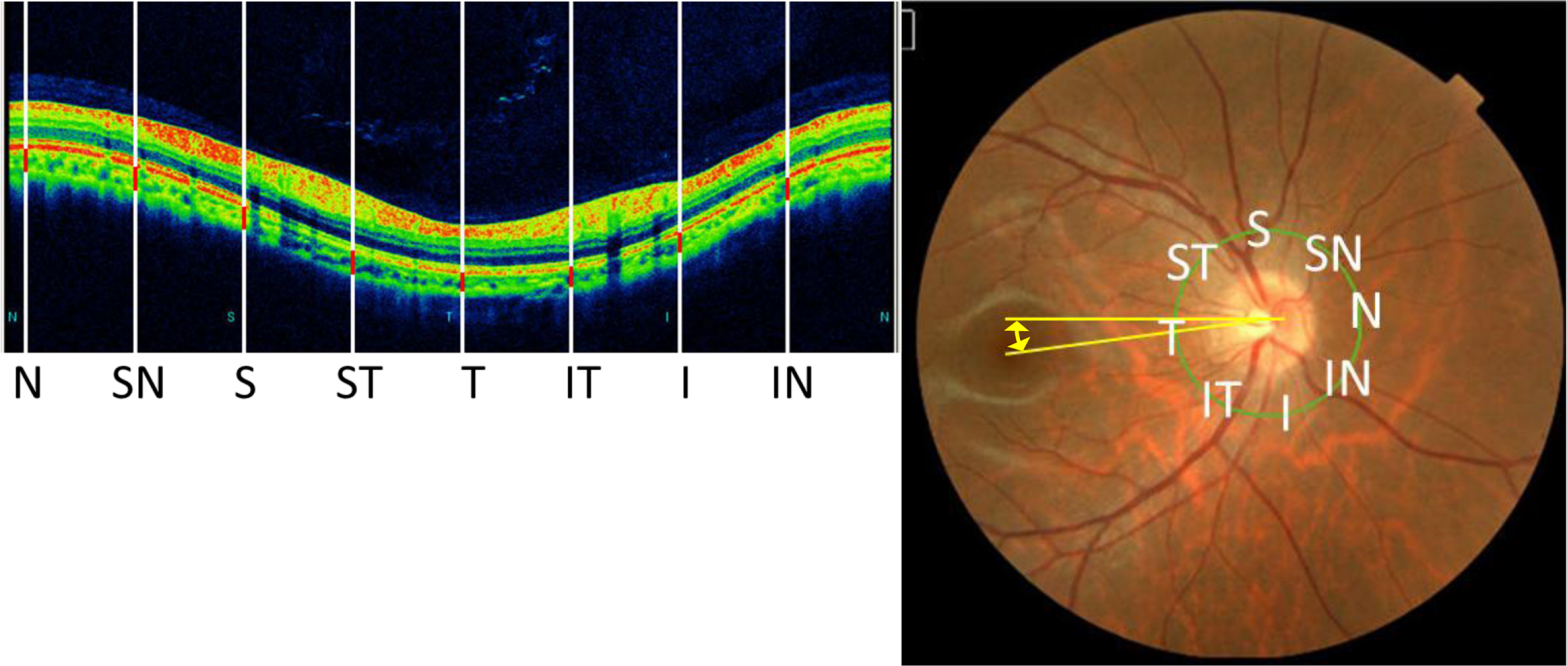
Technique used to measure the peripapillary choroidal thickness (ppCT). Optical coherence tomographic image (left) and fundus photograph (right) showing the eight circumpapillary sites where the ppCT was measured. To determine the zero coordinate of the circumpapillary circle, a line was drawn from the fovea to the center of the optic disc. The intersection of this line with the circumpapillary circle was designated as the zero coordinate and the temporal site. The other 7 sites were set at 45° intervals from the zero coordinate. The papillo-macular position (PMP) angle was determined in the fundus photographs as the angle form by a horizontal line and the foveal-optic disc line (yellow double arrows). N, nasal; SN, superonasal; S, superior; ST, superotemporal; T, temporal; IT, inferotemporal; I, inferior; IN, inferonasal.

The thickness of the ppCT was measured manually with the embedded software as the perpendicular distance between the outer portion of the hyperreflective line corresponding to the RPE to the hyporeflective line or margin corresponding to the sclerochoroidal interface. The distance was marked manually by an experienced grader who was masked to the other ocular characteristics of the subject. The locations of the sites around the optic disc were: temporal, superotemporal, superior, superonasal, nasal, inferonasal, inferior, and inferotemporal sectors [14, 15]. Initially, the temporal ppCT site was determined to be at the intersection of a line connecting the center of the optic disc and the fovea in the color fundus photographs with the circumpapillary circle. The other measured positions were located at 45° intervals from the temporal site (Fig 1).

### Determination of papillo-macular position (PMP) and optic disc tilt

Color fundus photographs and OCT images were taken at the same time with the TOPCON 3D OCT-1000 MARK II. The PMP is the angle formed by a horizontal line and a line connecting the optic disc center and the fovea in the color fundus photograph (Fig 1 right) [29].

Degree of optic disc tilt was determined by our method reported previously [27]. In brief, the course of the retinal pigment epithelium (RPE) was marked on the OCT B-scan images manually. The ‘x’ and ‘y’ coordinates of each mark were determined automatically by the ImageJ program (Image J version 1.47, National Institutes of Health, Bethesda, MD, USA). The ‘x’ and ‘y’ coordinates of each pixel were converted to a new set of ‘x’ and ‘y’ coordinates with zero at the center of the wave. Finally, the converted data were fit to a sine wave equation (*y* = *a* × sin(b× x−c)) with the curve fitting program of ImageJ. The ‘a’, ‘b’, and ‘c’ are constants calculated by the least squares method of the curve fitting program of ImageJ. The constant ‘a’ is the amplitude of the sine wave, and a larger ‘a’ will make the amplitude of the wave larger and make the optic disc tilt greater. Because the eyes were relatively stationary throughout the measurements, the retinal plane of macular area was fixed at an orientation approximately perpendicular to the reference light axis. Then, the optic disc tilt examined was represented by the angle between the optic axis and the optic disc tilt. The amplitude of the sine wave, ‘a’, was considered to represent the degree of the optic disc tilt relative to the optical axis [27]. The eyes were stationary throughout the measurements, and the retinal plane of the macular area was vertical to the optical axis of the eye.

### Statistical analyses

All statistical analyses were performed with the SPSS statistics 19 for Windows (SPSS Inc., IBM, Somers, New York, USA) and the statistical programming language R (version 3.0.2, The R Foundation for Statistical Computing, Vienna, Austria). Steel-Dwass multiple comparison tests were used to compare the ppCT at the different sectors. The relationships between the ppCT and the axial length, optic disc tilt, and PMP were determined by Spearman’s correlation analyses. Multiple regression analyses were also used to determine the association of the ppCT and the same factors because each of these factors was significantly correlated with the ppCT. A *P* <0.05 was taken to be statistically significant.

## Results

One hundred and thirty-three young Japanese volunteers were initially examined. Seven eyes were excluded because of ocular diseases or prior ocular surgery, three eyes because of superior segmental optic hypoplasia, one case because of glaucoma, and three cases because of prior laser-assisted *in situ* keratomileusis. Twelve other eyes were excluded because of difficulty in identifying the sclerochoroidal interface. In the end, the right eyes of 114 individuals were studied. The demographic information of the participants is presented in Table 1. The mean ± standard deviation of the age was 25.8 ± 3.9 years, and the mean refractive error (spherical equivalent) was -4.79 ± 3.31 diopters. The mean axial length was 25.46 ± 1.43 mm. The mean PMP angle was 5.43 ± 3.47 degrees, and the mean optic disc tilt was 53.08 ± 23.93 pixels. Raw data in this study was available in S1 Table.

**Table 1:** Demographic information of the participants.

Demographics	Mean ± SD	Range
**Age (years)**	25.8 ± 3.9	22 ~ 39
**Sex (men/women)**	79 / 35	
**Refractive error (spherical equivalent; D)**	-4.79 ± 3.31	-14.25 ~ 0.50
**Axial length (mm)**	25.46 ± 1.43	22.38 ~ 30.42
**PMP (degrees)**	5.43 ± 3.47	-3.14 ~ 13.27
**Optic disc tilt (pixels)**	53.08 ± 23.93	13.41 ~ 110.07

SD; standard deviation, PMP; papillo-macular position

**Fig 2:**
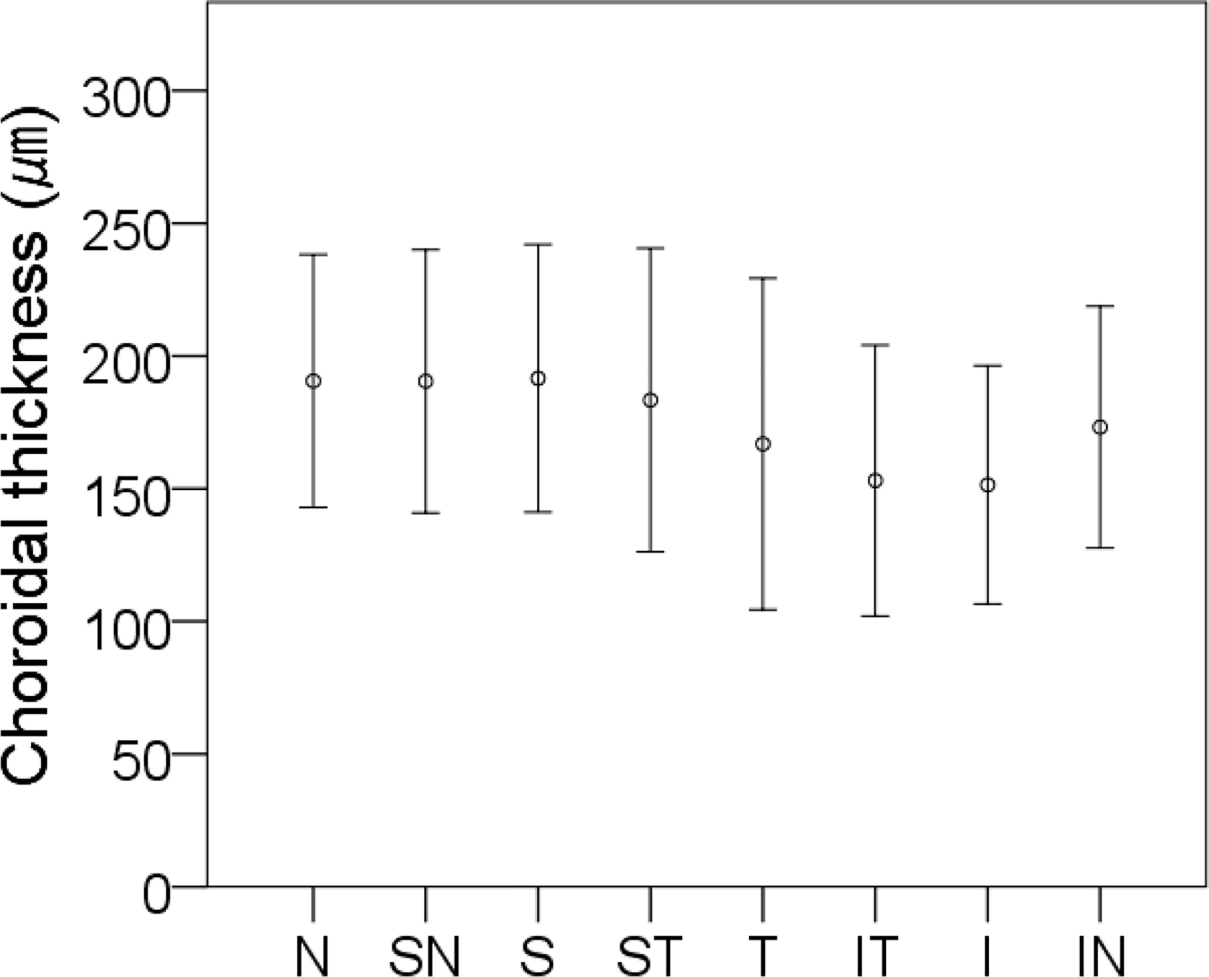
Means ± standard deviations of the ppCT in eight locations. N, nasal; SN, superonasal; S, superior; ST, superotemporal; T, temporal; IT, inferotemporal; I, inferior; IN, inferonasal.

The means ± standard deviations of the thicknesses of the ppCT in the nasal, superonasal, superior, superotemporal, temporal, inferotemporal inferior, and inferonasal sectors were 190.62 ± 47.66 μm, 190.50 ± 49.59 μm, 191.59 ± 50.44 μm, 183.41 ± 57.19 μm, 166.87 ± 62.45 μm, 153.09 ± 51.10 μm, 151.49 ± 44.58 μm, and 173.21 ± 45.58 μm, respectively. The thickness of the ppCT in the inferotemporal and inferior sectors were significantly thinner than that of the nasal, superonasal, superior, superotemporal, and inferiornasal sectors (all *P* <0.05; Fig 2).

**Fig 3:**
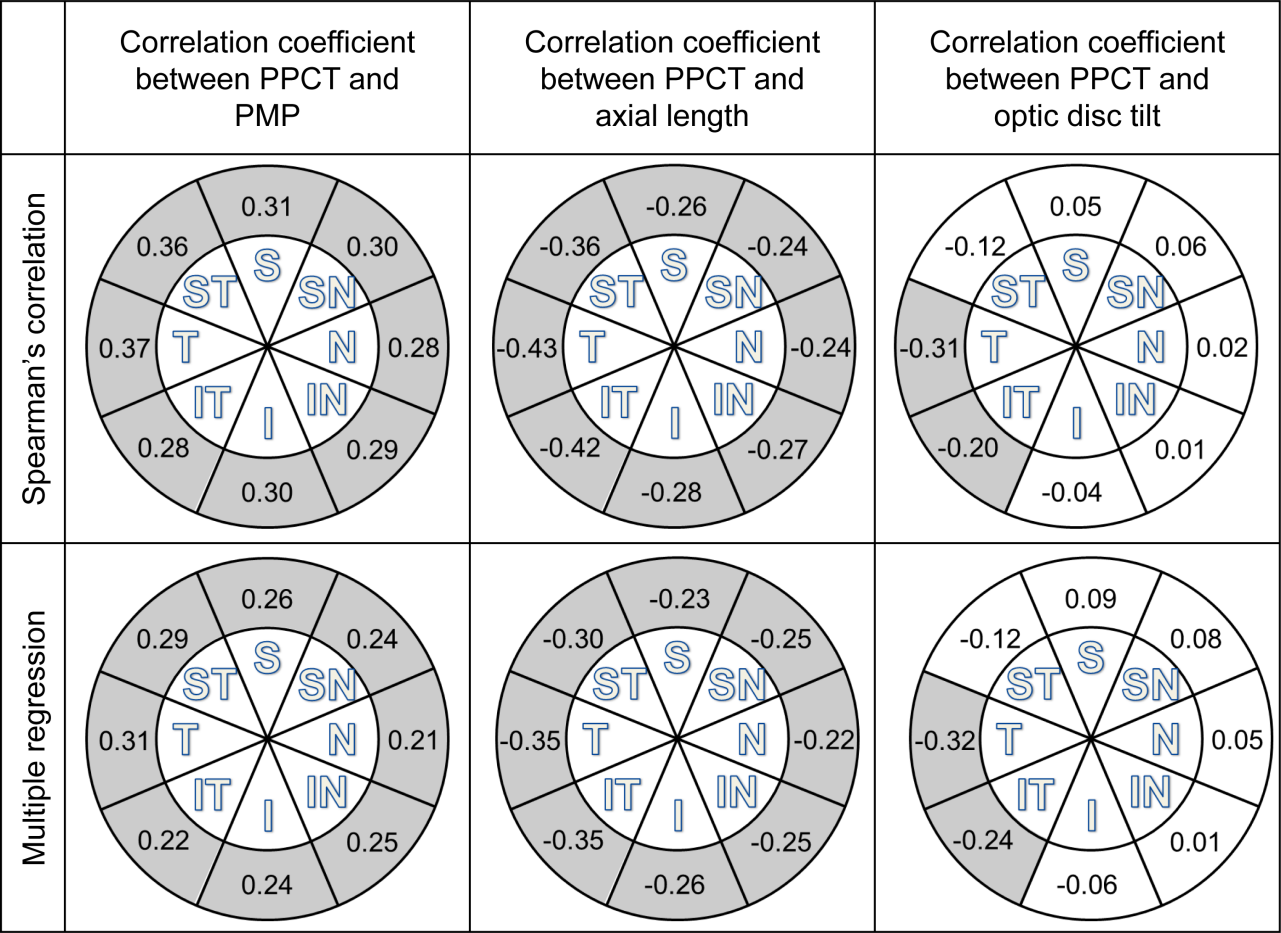
Spearman’s coefficients of correlation and standardized coefficients of multiple regression for the PMP, axial length, optic disc tilt, and the ppCT. Sectors that are significantly correlated are shown in gray. ppCT, peripapillary choroidal thickness; PMP, papillo-macular-position; N, nasal; SN, superonasal; S, superior; ST, superotemporal; T, temporal; IT, inferotemporal; I, inferior; IN, inferonasal sectors.

The Spearman’s coefficients of correlation and the standardized coefficients of multiple regression for the axial length, optic disc tilt, and PMP angle and the ppCT are shown in Fig 3. For both types of regression analyses, all locations of the ppCT were significantly and positively correlated with the PMP (r = 0.21 to 0.37, *P* <0.05 for all). The ppCT was also significantly and negatively correlated with the axial length (r = -0.43 to -0.22, *P* <0.05). For both types of regression analyses, the optic disc tilt was significantly and negatively correlated with the ppCT of the temporal and infrotemporal sectors (r = -0.32 to -0.20, *P* <0.05), but not with the other locations (r = -0.12 to 0.09, *P* ≥0.05).

## Discussion

Our results showed that the ppCT in all sectors was thinner in eyes with longer axial lengths. This finding is consistent with the results of earlier studies that reported that the average ppCT is thinner in eyes with longer axial lengths [14, 15]. This is probably because the sclera, choroid, and retina are stretched during the elongation of the axial length resulting in a decrease of the ppCT.

Importantly, the ppCTs of the temporal and inferotemporal sectors were thinner in eyes with a greater tilt of the optic disc but not in the other sectors. This has not been reported. Considering clinical cases, eyes with a long axial length are not always spherical but more frequently oval with a long antero-posterior axis [30]. In these eyes, the optic disc and nerve are often tilted temporally away from the visual axis. Thus, the retino-choroidal tissue can be stretched more strongly on the temporal side than on the nasal side. As a result, the temporal ppCT might become thinner than the nasal ppCT.

To examine the relationship between the ppCT sectors, we calculated the ratio of ppCT of the temporal sector to the mean ppCT of the other sectors, i.e., the temporal ppCT ratio = temporal ppCT/mean ppCT of all other sectors. The results showed that the average ratio ± SD was 0.93 ± 0.19 with a range from 0.41 to 1.32. This ratio was significantly and negatively correlated with the axial length (r = -0.43, P <0.001), positively correlated with PMP (r = 0.23, P = 0.01), and negatively associated with the optic disc tilt (r = -0.56 P <0.001). These findings should be useful for showing the variations in the ppCT in the same eye.

**Fig 4:**
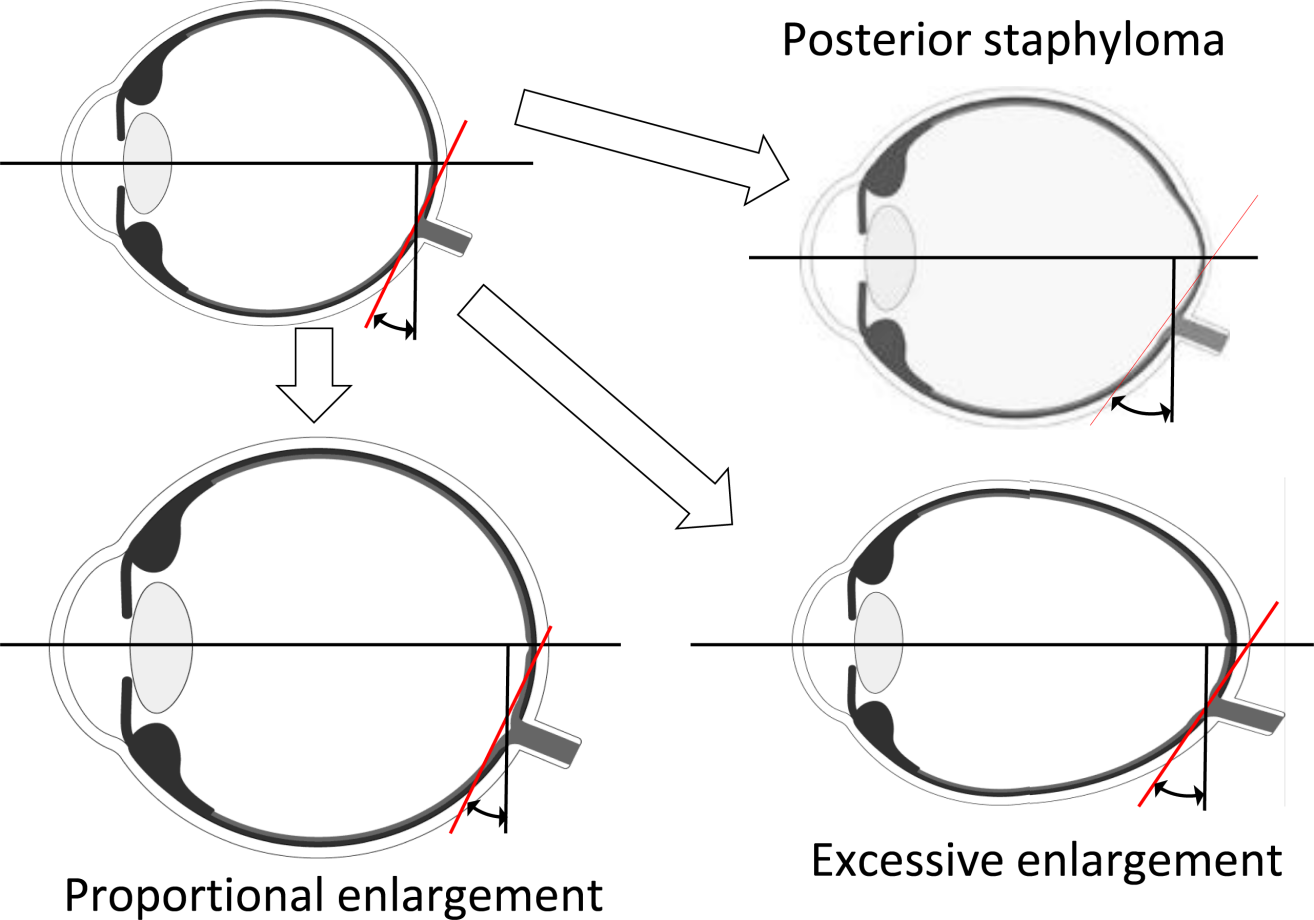
Relationship between ppCT and optic disc tilt. A proportional enlargement of the eyeball does not affect the optic disc tilt, but an excessive enlargement and subclinical posterior staphyloma will increase the optic disc tilt and stretch the temporal side of the optic disc like a conus.

In eyes with or without a posterior staphyloma, an elongation of the axial length may enhance the optic disc tilt and stretch the temporal side of the optic disc (Fig 4). Previous studies have found that the presence of a posterior staphyloma was significantly associated with choroidal thinning in highly myopic eyes [5, 14, 31]. A tessellation of the fundus was also significantly associated with choroidal thinning in myopic eyes [32]. In the young subjects of this study, an obvious posterior staphyloma was rare, but subclinical staphylomas were not rare in our earlier study [33]. Furthermore, a choroidal thinning in the inferolateral area of the optic disc was most common suggesting that the retina is stretched in this area. So even though a posterior staphyloma was not yet evident, an asymmetrical deformation of the peripapillary optic disc tissue might be at an early stage. This asymmetrical enlargement may be related to the present findings.

The PMP angle was also significantly and independently correlated with the ppCT in all sectors. Thus, the ppCT thickens when the fovea is located at a position inferior to the horizontal line. This is the first study to investigate the relationship between the ppCT and the PMP angle. Jonas et al reported that the distance between the optic disc and fovea was shorter when the fovea was located below the horizontal line [34]. This is consistent with the present finding that the ppCT is thicker in eyes in which the fovea is located inferior to the horizontal line. In these eyes, the foveo-papillary distance is shorter, and the stretching of the choroidal tissue would be slight. This would result in a thicker ppCT. The PMP angle and optic disc tilt would represent the relative three-dimensional optic disc position against the visual axis.

Gupta et al compared the mean ppCT in the different sectors of highly myopic eyes with a mean axial length of 27.23 mm to emmetropic eyes with a mean axial length of 22.03 mm. In both groups, the ppCT was thinnest inferiorly but in the myopic group, the ppCT was thinnest in the inferior and inferotemporal sectors [14]. In our study, the mean axial length was 25.46 mm, and the ppCT was significantly thinner in the inferotemporal and inferior sectors than the nasal, superonasal, superior, superotemporal, and inferiornasal sectors as reported earlier. All sectors of the ppCT in the highly myopic group were thinner than that in the emmetropic group, and the difference of the ppCT between the two groups was larger in the inferotemporal and temporal sectors than in other sectors. Although the optic disc tilt was not investigated in the study by Gupta et al, this big difference in the inferotemporal and temporal ppCT between the high myopic group and emmetropic group may be related to the optic disc tilt because most optic discs tilt toward the inferotemporal or temporal angle [26].

In our earlier study, it was found that the RNFL was thicker in eyes with a greater optic disc tilt [35]. So, we hypothesized that the ppCT is thicker in eyes with a greater optic disc tilt. However, the results of the current study showed that the temporal and inferotemporal ppCT sectors were thinner in eyes with a greater tilt of the optic disc. This was probably because the temporal and inferotemporal choroid was stretched more in these sectors by the optic disc tilt. This is supported by the fact that a conus in the temporal and inferotemporal sectors is often associated with optic disc tilting.

We examined the relationship between the parapapillary atrophy (PPA) and ppCT. The results showed that the ppCT was significantly thinner in eyes with PPA (82 eyes) than those without PPA (37 eyes). Specifically, the P value of each sector was; supratemporal (P = 0.001), temporal (P <0.001), inferotemporal (P <0.001), nasal (P = 0.03), supranasal (P = 0.11), inferior (P = 0.049), and inferonasal (P = 0.03, Mann-Whitney). Thus, the ppCT was thinner in eyes with PPA than those without PPA which was more evident in the supralateral and lateral sectors.

The data of the men were analyzed separately from that of the women. In the 81 eyes of men, the ppCT of every sector was thinner in eyes with longer axial lengths (r = 0.30 to 0.40, P <0.01). As the optic disc tilt increased, the ppCT of the temporal sector (r = -0.34, P = 0.002) and supra-temporal sector (r = -0.23, P = 0.04) decreased, but it did not do so in the other sectors. These results were the same as that of the overall group. In the 38 eyes of women, there was a tendency for the ppCT to be thinner in eyes with longer axial lengths, but the relationship between the ppCT and axial length was not statistically significant for any sector (r = -0.20 to 0.01, P >0.05). Thus, the ppCT was thinner in eyes in which the PMP angle was smaller (r = 0.14 to 0.33, P >0.04), but statistical significance was noted only in temporal sector. We conclude that the optic disc tilt was not significantly associated with the ppCT. The major reason for the ppCT not having been affected by other factors in women is the comparatively small number of participants. Another possibility was that the ppCT of women is less affected by axial length than that of men because the correlation coefficient between above two variables was significantly smaller in women than men. The difference in the distribution of the sexes is a limitation of this study and the further studies with the equal number of sexes is needed.

There are several other limitations in this study. There are many factors which can affect the ppCT, e.g., central corneal thickness, and intraocular pressure [14, 15]. None of these were included in our analyses. How these other factors affect the relationship between the localized ppCT and the PMP angle or the optic disc tilt must be examined in future studies.

The long-term goal of these studies was to determine the effects of these factors on the RNFLT. To examine the effects of these myopic factors, it was first necessary to study eyes with a wide range of refractive errors to obtain baseline values. The Japanese population is known to be the most myopic population in the world [36]. As a result, most of the subjects were myopic. This may have affected the results. On the other hand, the reliability of the examination was very high because no pathological factors such as cataract or vitreal opacities were present in young healthy individuals and the understanding of the examination was high in the present examinees. In addition, the narrow range of age can prevent the interference of cohort effects and aging effects.

In conclusion, our results indicate that the axial length, PMP angle, and the optic disc tilt are the major factors which can independently affect the ppCT. Therefore, these values must be taken into consideration when interpreting the ppCT of each patient. By doing that, a structure-function map can be made and the structural and functional measurements can be correlated more accurately. Knowledge of the normal ppCT and its profile may aid in understanding the pathophysiological changes in eyes with glaucoma and myopia better.

## Supporting information

S1 Table Raw data. (XLSX)
